# Cattle mortality as a sentinel for the effects of ambient air pollution on human health

**DOI:** 10.1186/2049-3258-73-S1-P22

**Published:** 2015-09-17

**Authors:** Bianca Cox, Antonio Gasparrini, Boudewijn Catry, Frans Fierens, Jaco Vangronsveld, Tim Nawrot

**Affiliations:** 1 UHasselt, Diepenbeek, Belgium; 2 London School of Hygiene & Tropical Medicine (LSHTM), London, United Kingdom; 3 Scientific Institute of Public Health (WIV-ISP), Brussels, Belgium; 4 Belgian Interregional Environment Agency (IRCEL-CELINE), Brussels, Belgium; 5 Hasselt University, Diepenbeek, Belgium

## Background

Air pollution is a trigger for human mortality. Although studies of spontaneous animal disease can provide additional insights, the short-term effects of air pollution on mortality have never been studied in animal populations. Animal sentinels are less subject to concurrent exposures, bias due to confounding, and exposure misclassification than human populations. Therefore we investigated the association between ambient air pollution and the risk of mortality in dairy cows.

## Methods

We collected ozone (O_3_), particulate matter (PM_10_), and nitrogen dioxide (NO_2_) concentrations at municipality level for 87,108 dairy cow deaths in Belgium from 2006 to 2009. We combined a case-crossover design with distributed lag nonlinear models in the warm and cold period of the year.

## Results

We found acute and delayed effects of air pollution on dairy cattle mortality during the warm season. The increase in mortality for a 10 μg/m^3^ increase in 2-day (lag 0-1) O_3_ was 1.3% (95% CI: 0.3, 2.2), and the corresponding estimates for a 10 μg/m^3^ increase in same-day (lag 0) PM_10_ and NO_2_ were 1.2% (95% CI: -0.3, 2.8) and 9.4% (95% CI: 6.4, 12.4), respectively. Compared to the acute effects, the cumulative 26-day (lag 0-25) estimates were considerably larger for O_3_ (3.6%; 95% CI: 0.4, 6.9) and PM_10_ (5.1%; 95% CI: 0.8, 9.5), but not for NO_2_ (2.9%; 95% CI: -4.1, 10.3). We did not find consistent evidence for air pollution effects during the cold period.

## Conclusions

Our study in cattle adds to the epidemiologic findings in human populations and further improves its causality. Furthermore, our results indicate that air pollution effects go beyond short-term mortality displacement. Compared with human studies, we observed higher mortality risk associated with air pollution among cattle, suggesting that dairy cows may be sensitive indicators of air pollution and provide an early warning system for public health intervention.

